# Implementation of risk assessment process for breast cancer risk in primary care

**DOI:** 10.15406/jcpcr.2024.15.00552

**Published:** 2024-06-28

**Authors:** Sarah Tucker Marrison, Caitlin G Allen, Kevin Hughes, Holly Raines, Mattie Banks, Travita Lee, Kiersten Meeder, Vanessa Diaz

**Affiliations:** 1 Department of Family Medicine, Medical University of South Carolina, USA; 2 Department of Public Heath, Medical University of South Carolina, USA; 3 Department of Surgical Oncology Medical University of South Carolina, USA

**Keywords:** cancer screening, cancer risk assessment, breast cancer, genetic risk, telehealth

## Abstract

**Background:**

Current cancer prevention guidelines recommend assessing breast cancer risk using validated risk calculators such as Tyrer-Cuzick and assessing genetic testing eligibility with NCCN. Women at high-risk of breast cancer may be recommended to undergo additional or earlier screening. Risk assessment is not consistently implemented in the primary care setting resulting in increased morbidity and mortality in unidentified high-risk individuals.

**Methods:**

A single-arm interventional study was conducted in an academic primary care clinic for women 25–50 years old presenting for primary care appointments. Pre-visit workflows evaluated breast cancer risk using the Cancer Risk Assessment (CRA) Tool and information was provided to the clinician with guideline-based recommendations. Post-visit questionnaires and chart review were conducted.

**Results:**

The survey response rate was 24.5% (144/587) with 80.3% of responses completed online (94/117). The average age of respondents was 35.8 years with 50.4% White and 35.9% Black. There were no differences in response rate based on race. Risk discussion was documented in the medical record in 15.4% of cases with a higher rate of documentation in high-risk patient based on risk assessment as compared with average risk respondents (34.6% vs. 9.7%, p<0.01). In the high-risk women identified 11.4% (4/35) were seen by the high-risk breast clinic, and 5.7% (2/35) were referred for genetic evaluation. None had previously obtained MRI screening or genetic testing.

**Conclusions:**

There is limited identification and evaluation of women at high risk for breast cancer. Pre-visit surveys can be used as a tool to assess breast cancer risk in the primary care setting; however additional strategies are needed to implement systematic risk assessment and facilitate appropriate treatment based on risk level.

## Introduction

Breast Cancer (BC) is the second leading cause of cancer-related death among women nationwide.^1^ Evidence-based guidelines recommend tailored approaches for early detection, based on individual risk.^2–6^ Women at elevated risk for BC (>20% lifetime risk), based on validated screening tools (e.g. Tyrer-Cuzick), benefit from more aggressive screening to promote early detection of BC.^2,3^ Risk factors include family history, genetic mutations (e.g., BRCA1/2), hormonal therapies, body mass index, and breast density. For these individuals, recommendations may include screening at a younger age and use of additional screening modalities (e.g. MRI, genetic testing) to promote earlier cancer detection and improved overall survival.^3,4,7,20^

Supplemental MRI screening significantly increases detection of BC with sensitivity of 90%, compared with 37.5% for mammography in BRCA carriers.^7^ Genetic testing for predisposing mutations can impact both cancer screening and prophylaxis recommendations, as women with BRCA mutations have up to a 70% risk of BC.^8–10^ Importantly, other high- and moderate-risk genes are also common.

Although implementation into the primary care setting has been recommended as part of guideline- based care, uptake of risk-assessment has been limited, resulting in higher morbidity and mortality for unidentified high-risk individuals.^11,12^ Primary care physicians (PCP) acknowledge the value of screening for inherited cancer risk; however, they reported low use of and confidence in the ability to use validated risk assessment tools.^12–15^ Only 40% of PCPs report having ever used a risk assessment tool and only 8.6% reported confidence in their ability to use it to identify women at increased risk of BC.^13,14^ Previously identified barriers include inadequate time, insufficient training, lack of clinician knowledge, competing priorities, and lack of tools.^14–16^ When female primary care patients have been surveyed, 85% supported the statement that risk assessment was a good idea^17^; however, self-perception of risk is poor with only 10% of women endorsing an accurate risk perception of their breast cancer risk.^18^ Within primary care, clinical decision support systems (CDSS) have demonstrated improved outcomes.^19^ Breast cancer risk assessment tools increase the intention of patients to complete screening, but additional interventions are needed to improve screening completion.^20^ Shared decision-making components to enhance uptake of BC screening include:

- use of clearly understandable information,
- inclusion of personal risk factors,
- discussion on the benefits and harms of screening
- clinician engagement.^21,22^

The use of clinic-based computerized intake systems (e.g. EHR) have been found to facilitate improved discussion on cancer risk.^23,24^

Previous studies in the United States and the United Kingdom have sought to evaluate proactive invitation for risk assessment of cancer risk with a 16–18% response rate, identifying a population of 10.6% of respondents in the primary care setting at an increased risk of BC.^24^ Uptake of risk assessment was limited in minority ethnic communities and individuals with reduced literacy. Some previous studies have utilized the time of mammography screening for risk assessment; however, this does not consider younger women who may be eligible for screening before the recommended screening age. Early mammography screening in high-risk women identified by cascade testing 40–49 was found to reduce mortality due to breast cancer by 12–29%.^17^ The current study evaluates the implementation of risk assessment information into the primary care setting, using the risk assessment tool combining both strategies of electronic health CDSS and physician-based discussion to support improved identification of high-risk individuals in the primary care setting. The study developed considers physician reported barriers to implementation of risk assessment and evaluates a technology-based approach to risk assessment evaluation in primary care.

## Material and methods

### a Semi-structured interviews

Provider level barriers to risk-stratified screening in primary care were evaluated. Eleven semi-structured interviews were conducted virtually with primary care providers in Medical University of South Carolina primary care clinics. Interviews were transcribed using Microsoft Teams. The interview template was developed with a focus on the Consolidated Framework for Implementation Research (CFIR) constructs of *intervention characteristics* to identify preferences and acceptability for strategies for intervention implementation including use of EHR data, algorithm-based screening, use of targeted visit types, pre-visit screening tools (e.g., CRA Health),^25^ and use of artificial intelligence tools. Following transcription, interviews were checked for accuracy. Coding was conducted by 2 separate individuals using rapid sequence qualitative analysis.

### b Intervention:

**Figure 1:**
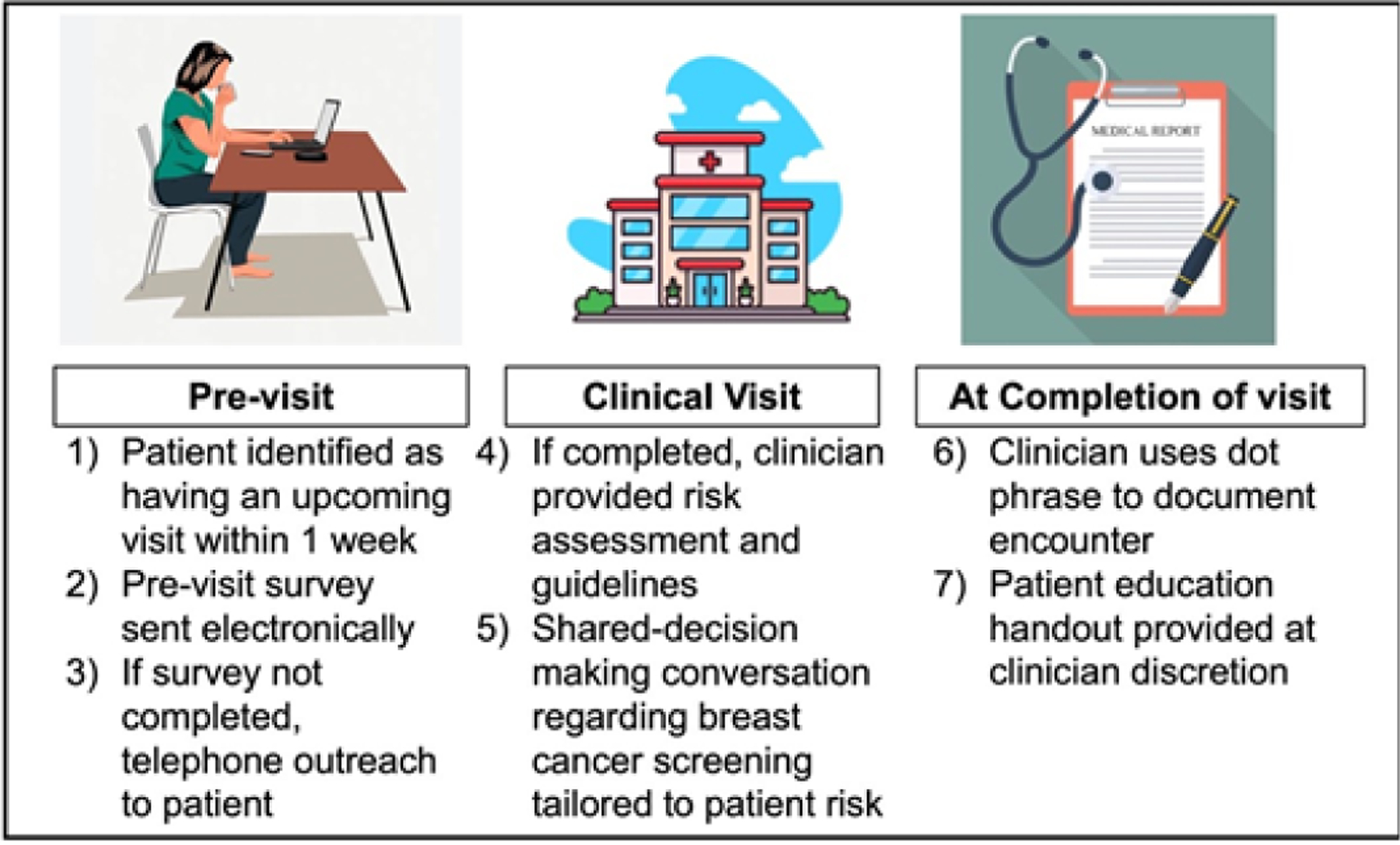
Intervention outline. Components of intervention based on timing relative to appointment.

A clinician in-training session was held on November 5^th^ with the full clinical staff and on November 11^th^ with clinicians only. A summary email with resources and intervention processes was sent to all clinicians, prior to intervention initiation. Women 25–50 years old, without a previous history of breast cancer presenting to a primary care visit from November 2022-May 2023 were sent a link to their e-mail with an invitation to complete an online survey assessing breast cancer risk using the CRA Tool. A dot phrase was created to document communication, including documentation of risk assessment, inclusion of appropriate guidelines, and information for coding individuals at increased risk. A follow-up link was sent to the e-mail of those who had not yet completed the survey. Beginning December 2022, an additional opportunity to complete the survey by phone was added. The CRA Tool evaluates Breast Cancer Risk using Tyrer Cuzick 6, Tyrer Cuzick 7, Tyrer Cuzick 8, BRCAPro, Gail, and Claus Risk Assessment and evaluates the eligibility for genetic testing by the NCCN guidelines. Risk information with guideline-based recommendation was provided to clinicians in advance of the scheduled appointment. Following the scheduled appointment, women who had completed the risk assessment, attended their scheduled appointment and were not opted out of research, were sent a REDCap link to complete a survey evaluating satisfaction and acceptability of the risk assessment tool and self-reported understanding of breast cancer risk. Intervention information is shown in (Figure 1). The project received MUSC Institutional Review Board (IRB) approval.

### c Chart review:

Following intervention completion, chart review was conducted to evaluate breast cancer screening and timing of screening including ultrasound, MRI, and mammography as well was referral to the high-risk breast clinic and genetic counseling. Data extraction was performed by 2 study personnel.

### d Statistical considerations:

Descriptive statistics were calculated for all variables. Group means and standard deviations are presented for continuous variable. Counts and percentages are presented for categorical values. Comparisons were made between groups using the student’s t-test. Patient level data collected included demographic data, social determinants of health, and barriers to accessing health care. Patient specific outcomes will include patient satisfaction, patient understanding, and technology acceptability.^26^

## Results

### a. Interviews on strategies and barriers for implementation of BC risk assessment:

Eleven interviews were conducted with primary care clinicians at the Medical University of South Carolina working in four different clinical settings. The average age of participants was 39 and 63.3% were female, 27.7% were male, and 9.0% nonbinary. Physicians identified a number of barriers to risk assessment in primary care including

1. Lack of training in BC risk assessment
2. Limited time during clinical visits
3. Need for integration of risk assessment in clinical workflows and the EHR.

Factors to promote increased uptake of risk assessment focused on the need for automation and integration within the EHR. The majority of participants were white (90.9%). All of the interviewed respondents reported that they would be willing to implement an EHR intervention for risk stratified breast cancer screening within primary care. Successful implementation would benefit from leadership and system level support as well as support from resources within the institution. (Table 1) summarizes findings from the qualitative analysis of the physician interviews. In the planning domain, intervention development considered workflows that reduced the clinical burden on the staff and automating available processes. Clinician education, with regard to both knowledge of the guidelines and the evidence behind the risks and benefits of enhanced screening protocol was requested. External incentives on the system and insurance side were found to contribute to motivation and resource allocation to complete screening. Additional themes identified that breast cancer screening is a priority for clinicians in their care for patients and that individual and system level benefits should be considered for sustainability of the intervention. Overall, breast cancer screening and implementation of risk assessment was found to be a priority and all interviewed participants felt they and their clinical setting would be agreeable to participate in an intervention to enhance screening.

### b. Screening rates:

The overall survey response rate was 24.5% (144/587). The majority were completed online (80.3%) with the remainder completed by telephone. In the first month, prior to initiation of follow up telephone calls, the response rate was 9.7%. Initiation of reminder calls increased screening rates; however, the majority of respondents were not reached by phone and did not complete the survey by telephone. In a subset of telephone contacts (n=82), 56.1% of phone calls had a voicemail left, 11.0% of patients indicated they would complete the survey online, 12.2% of patients were not able to be contacted, 8.5% opted out of research, 8.5% had an interval cancellation of their appointment, 2.4% encountered a technical error, and 1.2% completed the survey by telephone. Thirty-five (24.3%) high risk respondents were identified, of which 31 (21.5%) had a lifetime risk of breast cancer >20%. Twenty-six (18%) of respondents had indications for genetic testing for which only 7 (27%) self-reported completion. No high-risk respondents reported previously obtaining MRI screening. The demographic information for individuals offered screening, completing screening, and completing the redcap survey are shown in (Table 2). Differences in completion of the CRA survey were not observed based on race when White and Black and African American women were compared.

### c. Physician documentation and referral rates:

**Figure 2:**
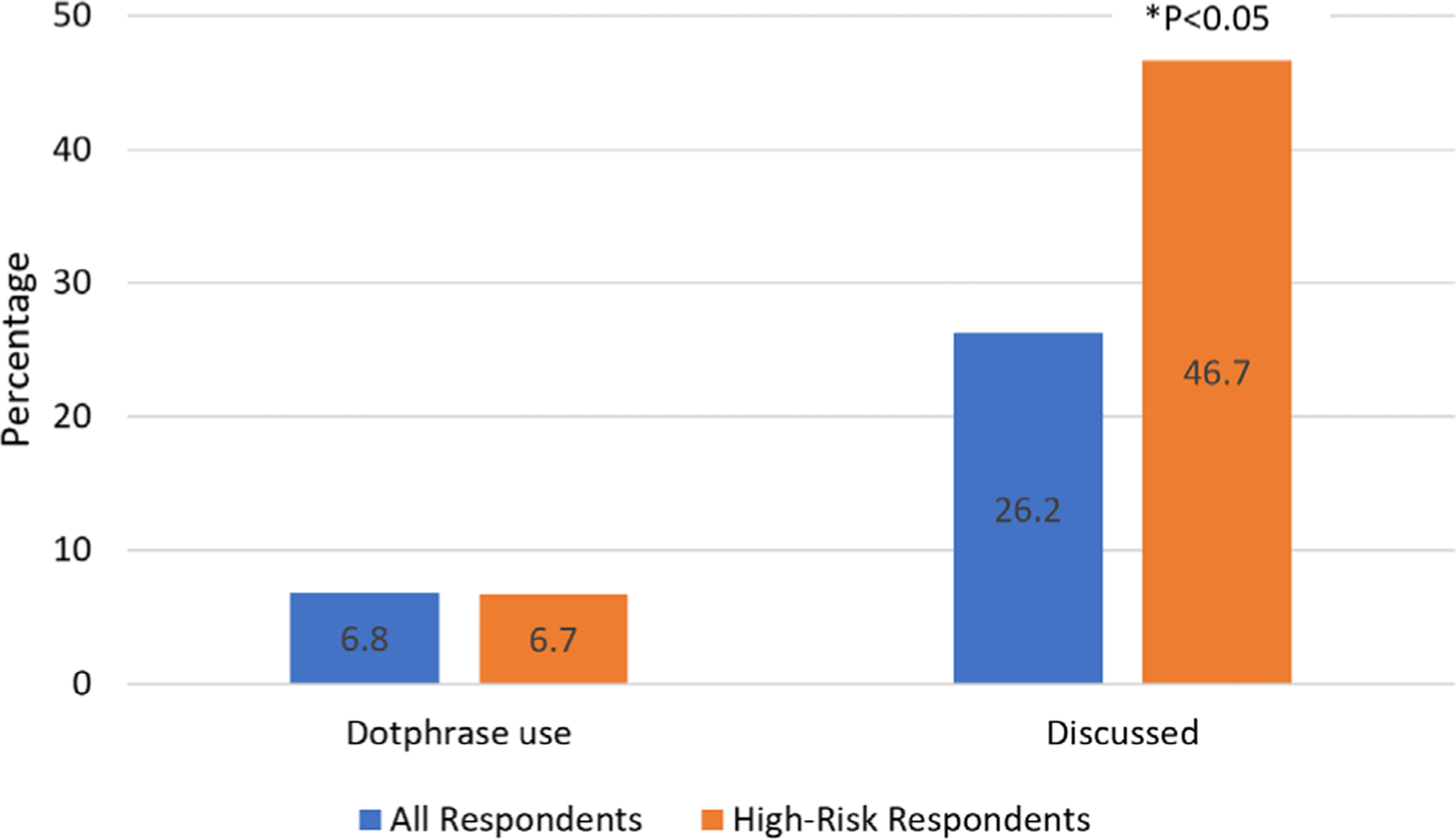
Documentation of risk discussion in women completing breast cancer risk assessment. Although dot phrase use was limited, in 26% of encounter, clinicians documented risk assessment discussion in 26% in a higher percentage of encounters in women identified as having an increased risk of breast cancer than average risk individuals.

Surveys were completed by 144 participants. Of these, there were 41 individuals for whom there was an appointment cancellation or no show. For the remaining appointments, dot phrase use was 6.8% and documented discussion was 27.2% (28/103) (Figure 2). Among high -risk respondents, there was a documentation rate of 46.7% (14/30). During the study period, 4 referrals were placed to the high-risk clinic (11.4% eligible), 1 to genetics, and 1 patient underwent genetic testing (5.7% eligible for genetic testing received testing or referrals).

### d. Patient acceptability, feasibility, and satisfaction:

**Figure 3:**
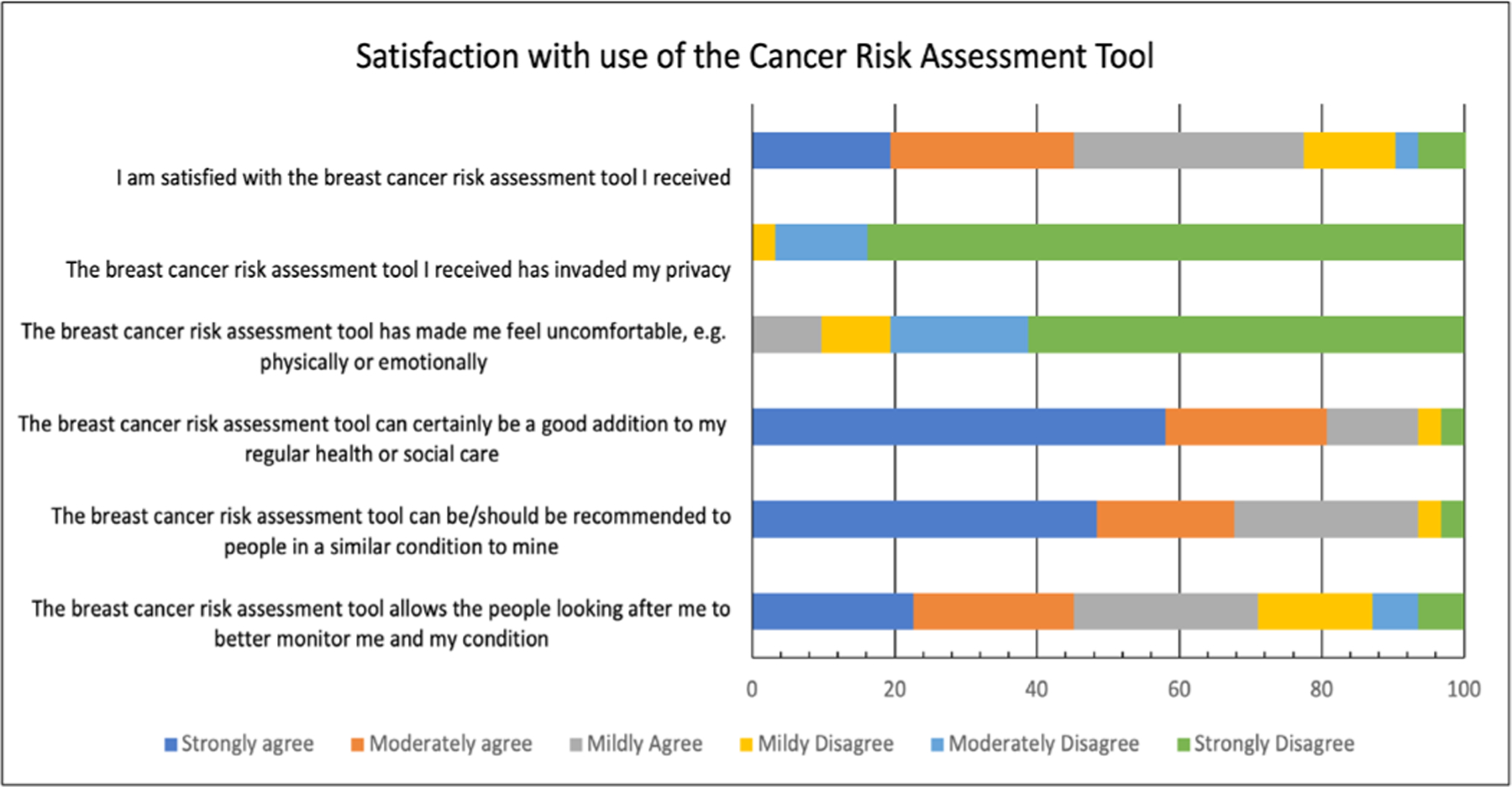
Evaluation of implementation of breast cancer risk assessment. Survey responses regarding acceptability of breast cancer risk assessment using the cancer risk assessment tool. Participants were surveyed following clinical visit.

When surveyed 42.5% (n=33) of REDCap survey respondents reported discussing the CRA risk survey with their clinician with 92.9% (n=14) reporting that their health care provider was good at explaining the reason for any medical testing. Overall, 85.7% of respondents reported they had a clear understanding of their breast cancer risk and 92.9% reported they had a clear understanding of their future plans for breast cancer screening. Patients did not report that it invaded their privacy or made them feel uncomfortable. Patients who discussed their risk results were more likely to report that the breast cancer risk assessment tool improved their health (9/12) than those who did not (4/14) (p=.018, chi-squared 5.57). Overall, majority of patients were satisfied with the breast cancer risk assessment tool. Opportunities were present to improve explanation of the tool can be considered in future interventions (Figure 3).

## Discussion

This study evaluated the barriers and facilitators to implementation of breast cancer risk assessment in the primary care setting and subsequent implementation of risk assessment in an academic primary care clinic. Barriers to implementation included the clinical burden, physician education, and limited tools and resources to implement risk assessment. Facilitators included employing out-of-visit strategies to limit clinical burden, automation using EHR tools, incorporation of the entire clinical team and leadership, and the addition of clinician training opportunities. These barriers identified align with the findings of previous studies including by Spalluto et al. who identified domains of knowledge and understanding, workflow, and personnel limitations with the concern of limitations of physician clinical time.^27^ Within our study, there was an increased focused in interviewed respondents on use of EHR tools and automation to support risk-assessment process. Consistent with previous studies, our study identified the need for support and inclusion of the entire clinical team during the development and implementation process. This feedback was incorporated in the implementation intervention through an in-training session attended by clinicians and clinical team members, utilization of a pre-visit workflow, development of EHR smart tools, and provision of additional resources to facilitate billing and coding. Future implementation interventions should consider further integration into both the clinical workflow and EHR to reduce time barriers to implementations. Ongoing educational efforts are needed to promote further risk assessment in primary care including appropriate discussion of management options for women identified at increased risk for breast cancer.

Intervention implementation resulted in modest screening rate of 24.5%, utilizing only a pre-visit workflow. This is an increase from previous published studies in the primary care setting including by Qureshi et al. with a 16– 18% response rate. Based on previously published studies, the rate of high-risk patients is higher than the previously published rates of high-risk patients at approximately 10–13%.^24,28^ There was a response bias in respondents in our study concerned about their breast cancer risk with a high-risk rate of 18–19% as compared with previously identified population rates of 8–10%.^17,24^ Breast cancer lifetime risk varies based on age with decreasing lifetime risk with increasing age and some variability based on model used for risk assessment with the high percentage of individuals identified as a lifetime risk >20% with Tyrer-Cuzick.^29^ Based on the younger population selected for inclusion, the percent of respondents with lifetime risk >20% would be expected to be higher than previous evaluations. Additional in-clinic and post visit processes should be explored to improve response rate while still limiting clinical burden. Although the addition of phone calls improved uptake of the risk-assessment, majority were completed online and not over the telephone. As there is a tablet-based option to complete the CRA assessment, future interventions can add this option in addition to the pre-visit workflow. The response rate of white patients and black or African American patients was representative of the clinic population. Although the CRA risk assessment questionnaires are available in Spanish, the clinic population had limited representation from Hispanic and Latino patients and assessing implementation in this population was not feasible.

Thirty-five high-risk patients were identified, with limited uptake of guideline-based care (MRI screening and genetic testing). The intervention increased uptake of risk assessment, however additional opportunities exist to support risk-assessment documentation and follow up care. Risk-assessment discussions were reported and documented more often in high-risk individuals. Further evaluation should explore additional clinical knowledge and barriers to delivering guideline-based care. Ongoing education on management of high-risk individuals and additional EHR tools (i.e order sets, BPAs) can further support guideline-based management of individuals identified as high-risk. The rates in our study while low, are not inconsistent with the low rates reported in previous studies evaluating management of women at increased risk of breast cancer in primary care setting.^15^ Comprehensive approaches to risk-assessment supporting not only risk-assessment but also management will be needed to advance care to align with guideline-based recommendations.

Participants overall reported satisfaction with the process of risk assessment. Additional opportunities for further evaluation of the initial risk assessment survey would be beneficial. Our study expands upon the previous findings; demonstrating 85% of women consider risk assessment to be a good idea. In a pilot implementation phase, the majority of women reported that the breast cancer tool would be a good addition to their regular health care and that after use in and experience in the primary care setting (93.6%). Based on this response, patient feedback supports a broader implementation of risk assessment tools in the primary care population. In addition to acceptability of the screening process, patients reported a good understanding of their breast cancer risk and screening plan. Improvement in the explanation of outreach may be a potential opportunity to improve the questionnaire response rate. This could include expansion of the introduction of the screening process with the survey, patient messaging within the EHR providing education about the screening, and further integration within the electronic health record to improve capacity for patients to interact with the clinical team.

Limitations of the study include implementation into a single academic family medicine clinic. Further analysis into opportunities for expansion into other clinical settings including community-based clinic settings and underserved settings will be necessary to ensure work processes and implementation are appropriate. Additionally, the clinic while diversely providing care for white and black or African American patients does not provide representation for additional racial and ethnic minority populations. As a pilot study that was not powered to evaluate efficacy, with a focus on evaluating acceptability and feasibility, larger multi-site studies are needed to evaluate efficacy of implementation of risk assessment information.

## Conclusion

Implementation of guideline-based breast cancer risk-stratification within primary care and subsequent counseling of high-risk women on recommended increased screening and risk reduction options can decrease breast cancer morbidity and mortality. The single arm intervention provides a modest pre-visit survey response with further opportunities to improve risk-assessment in the primary care setting. Although EHR tools were developed, additional opportunities for automation can be considered to improve risk documentation and assessment.

## Figures and Tables

**Table 1:** Qualitative analysis of physician interviews on implementation of risk assessment in primary care using CFIR

Domain	Findings	Quotation	Intervention feature
Planning	Annual exams best screening opportunity	“It would be helpful if we could automate some of these things where the patient was given	Utilization of pre-visit work flow
	Work flow should limit clinical time Team based approach inclusive of entire clinical care team Automation using Electronic Health Record (EHR) tools	the [tool] they could just actually go through the tool themselves. But prior to me coming in and that would be really helpful.”	Development of documentation smart phrase
		“The more that can be done outside of clinic or the less work it puts on the nurses the better.”	
**Characteristics of the individual**			
Self-efficacy	Variability in clinician knowledge	“I think just having that first, the best approach would just to have knowledgeable providers and I will admittedly say my knowledge beyond just basic standardized guidelines at like the USPS TF level. My knowledge there is pretty limited.” “The other thing that I just think is incredibly important is education of the faculty of the	Clinician-in training session held
	Benefit of clinician training resources Need for provision of resources for management and referral	providers there about what should be the next step.”	Development of patient and physician educational handouts
**Outer setting**			
External policies and incentives	Breast cancer screening aligned with accountable care organization incentivized priorities	“Breast cancer screening is a ACO metric, and so I think if you automated it so that it didn’t require a whole lot of extra work on the provider standpoint, I think people would be really on board with it.”	Future opportunity to work with clinical care coordinators
Patient needs and resources	Patient barriers identified to screening include transportation and insurance	“I stopped ordering MRIs myself and just started referring to high risk breast clinic because of the insurance coverage. If I order it versus if they I send them to high-risk breast clinic, they tend to have better success at getting that stuff covered.”	Billing codes and diagnoses provided with the dot phrase created to facilitate improved coverage
**Inner setting**			
Relative priority	Breast cancer screening is a priority for clinicians	“I think we can all improve our, our breast cancer screening methodologies. And I think that this really does lay at the, at the, the hands of either primary care providers, or OB GYN’s to make sure that we’re doing it. And so, I think we could all improve our ability to better screen for breast cancer and screen appropriately by using risk stratification. I think that makes a lot of sense to reduce both, you know disease burden as well as you know, early mortality. I think both would be helpful if we had some sort of intervention like this.”	
Organizational incentives and other rewards	Focus on benefit both to individuals and the health system	“I think that MUSC in particular would be OK with this EHR driven thing as long as it didn’t cost the system more money than it benefited the system by increased screening”	Future opportunity for cost analysis of screening with associated implementation
Tension for change	Variability in multiple guidelines available	“I feel like we’re aimlessly doing breast cancer screening and I’ve felt that way for quite some time that there’s conflicting recommendations ..	
	Motivation to improve breast cancer screening processes	. And it seems like it could be something that would be so much easier to cohesively put in some type of risk stratification tool”	
Compatibility	Align with existing work flow processes including best practice advisories	“I think it could be done for sure . . .but I think again in in breast cancer risk assessment, something we’re doing a lot, just not as in depth as doing a screening tool, but I feel like it could definitely be done.”	
Implementation climate	Requires leadership and system support	“I think MUSC in general is very welcoming to anything that will make our lives easier and better for patients. So, I think certainly my experience with MUSC would lend to us adapting it very well.”	
	Requires additional institutional support including high-risk breast clinic		
Evidence strength and quality	Opportunity for improved education of clinicians	“I think really first it would be like I would want to know before I investing like the effort into that and I would just want to know that it does make a difference.”	Clinician-in-training session conducted as well as training session for all clinical team members
**Intervention characteristics**			
Adaptability	Adaptation to limited clinical time	“It just needs to be super user friendly. ..It’s just making sure that it’s simple so that people want to do it because I know the more clicks there are, the longer something is. The less likely someone is to complete it.”	Cancer Risk Assessment using super brief survey was employed rather than more detailed surveys available
	User friendly interface		
Design quality and packaging	Use of Electronic Health Record (EHR) smart tools beneficial	“I think a dot phrase that I could enter directly would be helpful. Alternatively, if that wasn’t possible, if there was a link or something, either through Epic”	Dot phrase was created to facilitate documentation

**Table 2:** Demographic information

	Non-respondents (n=443)	Respond to CRA (n=144)	Respondents to redcap (n=33)
Age	36.7 (7.4)	36.0 (3.0)	35.6 (7.9)
Race	53.4% White	51.3% White	66.7% White
	34.7% Black or African American	43.2% Black or African American	18.2% Black or African American
Hispanic or Latino	0%	3.20%	0%
Office visits/year	3.7 (3.5)	4.0 (4.0)	
Mammograms ordered >40	75.6% (n=148)	61.4% (n=53)	
Mammograms completed >40	76.90%	81.30%	72.7 (n=11)
